# A population-based national estimate of the prevalence and risk factors associated with hypertension in Rwanda: implications for prevention and control

**DOI:** 10.1186/s12889-017-4536-9

**Published:** 2017-07-10

**Authors:** Marie-Rosette Nahimana, Alypio Nyandwi, Marie Aimee Muhimpundu, Olushayo Olu, Jeanine Umutesi Condo, Andre Rusanganwa, Jean Baptiste Koama, Candide Tran Ngoc, Jean Bosco Gasherebuka, Martin O. Ota, Joseph C. Okeibunor

**Affiliations:** 1 WHO Country Office, Kigali, Rwanda; 2 Ministry of Health, Kigali, Rwanda; 3 Rwanda Biomedical Center, Kigali, Rwanda; 4 CDC, Port-Au- Prince, Haiti; 5 WHO Regional Office for Africa, Brazzaville, Congo

**Keywords:** Non-communicable diseases, Hypertension, Epidemiology, Risk factors, Rwanda

## Abstract

**Background:**

Hypertension is a leading cause of cardiovascular diseases and a growing public health problem in many developed and developing countries. However, population-based data to inform policy development are scarce in Rwanda. This nationally representative study aimed to determine population-based estimates of the prevalence and risk factors associated with hypertension in Rwanda.

**Methods:**

We conducted secondary epidemiological analysis of data collected from a cross-sectional population-based study to assess the risk factors for NCDs using the WHO STEPwise approach to Surveillance of non-communicable diseases (STEPS). Adjusted odds ratios at 95% confidence interval were used to establish association between hypertension, socio-demographic characteristics and health risk behaviors.

**Results:**

Of the 7116 study participants, 62.8% were females and 38.2% were males. The mean age of study participants was 35.3 years (SD 12.5). The overall prevalence of hypertension was 15.3% (16.4% for males and 14.4% for females). Twenty two percent of hypertensive participants were previously diagnosed. A logistic regression model revealed that age (AOR: 8.02, 95% CI: 5.63–11.42, *p* < 0.001), living in semi-urban area (AOR: 1.30, 95% CI: 1.01–1.67, *p* = 0.040) alcohol consumption (AOR: 1.24, 95% CI: 1.05–1.44, *p* = 0.009) and, raised BMI (AOR: 3.93, 95% CI: 2.54–6.08, *p* < 0.001) were significantly associated with hypertension. The risk of having hypertension was 2 times higher among obese respondents (AOR: 3.93, 95% CI: 2.54–6.08, *p*-value < 0.001) compared to those with normal BMI (AOR: 1.74, 95% CI: 1.30–2.32, *p*-value < 0.001). Females (AOR: 0.75, 95% CI: 0.63–0.88, *p* < 0.001) and students (AOR: 0.45, 95% CI: 0.25–0.80, *p* = 0.007) were less likely to be hypertensive.

**Conclusion:**

The findings of this study indicate that the prevalence of hypertension is high in Rwanda, suggesting the need for prevention and control interventions aimed at decreasing the incidence taking into consideration the risk factors documented in this and other similar studies.

**Electronic supplementary material:**

The online version of this article (doi:10.1186/s12889-017-4536-9) contains supplementary material, which is available to authorized users.

## Background

Hypertension is one of the five leading causes of mortality in the world and a major risk factor associated with more than 40% of deaths related to cardiovascular and renal diseases [1–6]. Because of its asymptomatic nature, many people with the disease remain undiagnosed and untreated thus resulting in increased premature and sudden deaths due to direct or indirect complications [7]. According to 2010 WHO estimates, 22% of adults aged 18 years and above were hypertensive, and 9.4 million deaths were estimated to have been caused by hypertension, which is about 7% of the global burden of disease [8]. Across the WHO regions, Africa has the highest prevalence of high blood pressure with 30% of the people affected, while the lowest was recorded in the American Region [8, 9]. According to a systematic analysis conducted in 2014, prevalence of hypertension increased from 19.7% in 1990 to 30.8% in 2010 in Africa [10]. The changing epidemiology of hypertension is associated with the global economic development resulting in aging population in some societies, and changing lifestyle resulting in increased prevalence of obesity, alcohol and tobacco consumption and physical inactivity [11–13]. Worse still, the level of awareness, treatment and control of hypertension remain low in Africa, thus most of the affected persons are unaware of their status [10].

Like many other developing countries, Rwanda is in a phase of epidemiological transition. While communicable diseases remain the major causes of morbidity and mortality in the country, the increasing incidence of non-communicable diseases such as hypertension results in a double burden of diseases [14]. This pattern is associated with improvements in the socioeconomic status of the country in the last 20 years resulting in changes in lifestyle [15]. A study conducted in Bugesera district in 2007 estimated the prevalence of high blood pressure at 16.8% [16]; this study identified age, overweight, dietary intake and physical inactivity as risk factors associated with development of hypertension. [16]. A cross-sectional survey conducted at an urban tertiary education institution in Rwanda found that 36% of employees were hypertensive and demonstrated a low level of awareness among hypertensive participants [17]. Low awareness was also documented in a study conducted among hypertensive patients enrolled in the outpatient department of Kigali University Teaching Hospital [18]. The review of medical records showed that hypertension accounted for 2.5% of total admissions in Ruhengeri district hospital out of which 47.4% presented with severe hypertension. The review highlighted that alcohol consumption, diabetes mellitus, congestive heart failure were significantly associated with hypertension [19]. WHO estimated the number of deaths attributable to hypertension in Rwanda at 18/100,000 [20]. Available health facility-based data showed that the proportion of people consulting for high blood pressure increased from 1.9% in 2009 to 6.4% in 2014 in Rwanda [14].

The discrepancy in the prevalence of hypertension between the community and hospital-based data is wide [14, 16]. This can be attributed to the asymptomatic nature of the disease with many people not being aware of their status, not self-reporting and hence not captured in the health facility-based data. Given that the community data is a more reliable reflection of the true burden of any disease, we identified the need to analyze the nation-wide survey data collected from a representative sample to better estimate the prevalence of hypertension in Rwanda.

This study is a nationally representative study and provides population-based estimates of the prevalence and risk factors associated with hypertension in Rwanda. The findings will serve as baseline for monitoring the changing pattern of hypertension and its risk factors, and inform development of appropriate policies, strategies, planning and targeting of preventive public health interventions.

## Methods

### Study design and survey participants

We conducted a secondary analysis of primary data collected from a national population-based cross-sectional study to assess the risk factors for NCDs; the primary study was conducted using the WHO STEPwise approach to Surveillance of non-communicable diseases (STEPS) and took place in Rwanda from November 2012 to April 2013. The survey assessed the prevalence of risk factors for Non-Communicable Diseases (NCDs) in the country [21]. Study participants included people aged between 15 and 64 years residing in Rwanda, who participated in the STEPwise national survey.

### Description of STEPS survey

### STEPS sample size and method

The sample size was calculated using the formula N = Z^2^ P (1-P)/e^2^ and assumptions described by WHO STEPwise approach to non-communicable disease risk factor surveillance [22], where N = sample size, Z = level of confidence, P = baseline level of the selected indicator and e = margin of error. P was estimated at 0.50 (recommended by the STEPS survey guidelines when the estimated baseline is unknown), Z = 1.96 (at 95% Confidence Interval), e = 0.05, thus the estimated sample size was *N* = 1.96^2^ × 0.5(1–0.5)/0.05^2^ = 384. This basic sample size was adjusted for design effect for complex sample design (1.50), age-sex estimates, 15–64 age range (10 year intervals) and the required sample size was therefore *N* = 384* 1.5* 10 = 5760. Assuming a non-response rate of 20%, the final sample size was therefore adjusted upward to: 5760/0.8 = 7200.

A multi-stage cluster sampling method was used to select participants from the population based on information from the 2012 census. The three clusters were enumeration areas (EAs) (defined as villages in the Rwandan context), households and eligible participant within the household (Additional file 1). In the first stage, the list of all the villages in Rwanda was obtained from the National Institute of Statistics of Rwanda (NISR) and used as a sampling frame for random selection of EAs. Probability Proportional to Size (PPS) sampling method was applied to randomly select 279 EAs, thus the selection was done based on the size of EAs. [23]. In the second stage, the number of households to be sampled in each EA was determined by dividing the total sample size by the total number of EAs selected, thus the number of households in each EA came to 26. These households were then selected from each of the identified EAs, using the systematic random sampling method. The sampling interval for household selection in each village was determined by dividing the total households in a village by 26. In the third stage, one eligible participant (aged 15–64 years) was randomly selected from each household and enrolled into the survey. Eligible participants’ and household identification, name, sex and age were recorded on the electronic Kish sampling system built in personal digital assistant (PDA) and a random selection of the survey respondents in the household was made through the PDA and Kish selection method^1^ was used in case more than one household members were eligible for the interview [24–26].

### Data collection tools and approach

The WHO STEPS for NCD risk factors surveillance consist of three sequential steps namely 1) gathering information on sociodemographic characteristics and key risk factors using a uniform set of questionnaires; 2) physical measurements of blood pressure, height, weight and waist circumference; and 3) biochemical measurements of parameters such as fasting blood glucose, blood lipids etc. The WHO generic STEPS instrument was adapted to the Rwanda context and used to collect data for the STEPS survey. The questionnaire had 133 questions grouped into six sections, and included ten questions on hypertension. Survey teams, each comprising of one supervisor whose role was planning and checking the completeness of the questionnaire, three experienced nurses who conducted the interviews and, one laboratory technician who collected capillary and venous blood samples for quality control at the National Reference Laboratory collected the data for the study. One community health worker (CHW) was assigned to each survey team to assist with notification of the heads of the selected villages about the day and time when the survey team will visit their villages and guide the survey team to the selected villages and households. Prior to data collection, the teams were trained on the objectives, methods of the survey, and the tools for data collection were pre-tested. Data were collected using PDAs. Before leaving the participant’s residence, the data collection team reviewed responses for completeness and any missing information was appropriately updated. The information from the PDAs was backed up and uploaded onto a computer using e-STEPS which is a suite of software that support implementation of data collection using PDA, and epi-data software on weekly basis. Data quality was assessed on a weekly basis and feedback was sent to the survey supervisors in the field to correct any discrepancies. The data were edited and coded as per the WHO STEPS manual and entered into the WHO recommended EpiData software version 3.1,

Socio-demographic information collected included age, sex, residence (rural, semi-urban and urban as defined by NISR), level of completed education, marital status, and occupation. Variables assessed included height, weight, tobacco use, alcohol intake, physical activity, high blood pressure, and biochemical measurements such as blood glucose, total cholesterol and HDL cholesterol.

### Definition and measurement of variables

#### High blood pressure

High blood pressure was defined as a systolic blood pressure of more than or equal to 140 mmHg and/or diastolic blood pressure more than or equal to 90 mmHg [27] or currently taking antihypertensive medications An automated blood pressure machine (OMRON® digital device) was used to obtain the blood pressure readings [22, 28]. Three readings were taken 3–5 min apart after 15 min rest of the survey participant. As recommended by WHO, the average of the last two readings were calculated and used as the final blood pressure measurement.

#### Body mass index (BMI)

BMI was expressed as weight in kilogram/height in square meters (kg/m^2^). Weight and height were measured using Genesis weighing scales with a laser for measuring height. The survey participants were measured without shoes and wearing only light cloths. Respectively, height and weight were measured to the nearest whole centimeter and 0.1 kg. BMI was classified as <18.5 kg/m2 (lean), 18.5–24.9 kg/m2 (normal), 25.0–29.9 kg/m^2^ (overweight), and 30+ kg/m^2^ (obese) [29].

#### Biochemical measurements

The finger prick method was used to collect capillary blood sample after 8 h of fasting. For every tenth finger-prick test performed, a venous sample was taken for quality control at the National Reference Laboratory. Total cholesterol, high-density lipoprotein (HDL) and fasting blood glucose were measured using CardioChek PA (Glucose, Cholesterol, and HDL) [30].

Blood glucose was categorized as normal (<5.6 mmol/l), impaired fasting glycaemia (≥ 5.6 mmol/L and <6.1 mmol/l), and raised fasting blood glucose (≥ 6.1 mmol/l, or currently on medication) [31]. For total cholesterol, normal range was defined as <5.0 mmol/l. The level of HDL cholesterol was classified as normal (≥1.03 mmol/l for males, ≥ 1.29 mmol/l for females) and low (< 1 mmol/l or 40 mg/dl in males, and <1.3 mmol/l or 50 mg/dl in females) [22].

### Data analysis

We extracted and performed secondary data analysis on all participants in the STEPS aged 15–64 years. The level of education, marital status, occupation, alcohol consumption and physical activity and BMI were re-categorized from the original dataset to facilitate the analysis and results interpretation (Additional file 2).

The data were weighted by sample weights and analyzed using Stata version 13. Descriptive analyses comprising frequencies of high blood pressure according to sociodemographic characteristics and behavioral health risks among study population by sex and age; and analytical analyses were performed. The Pearson Chi-square test was used to test the independence between variables. The association between sociodemographic characteristics, behavioral health risks and hypertension were determined using bivariate and multivariable analyses methods. Furthermore, multivariate logistic regression was conducted for all variables of interest with *p*-value <0.05 in the univariate model. These variables are socio-demographic including age, sex, residence, marital status, education, occupation; behavioral variables such as tobacco use, alcohol consumption, physical activities, and metabolic risk factors such as BMI, blood glucose, total cholesterol and HDL cholesterol which are risk factors recommended for assessment in the STEPS survey manual [22]. Adjusted odds ratios (AOR), 95% confidence intervals (CI), and *p*-values were generated to determine independent predictors for hypertension.

## Results

Over seven thousand (7116) participants aged 15–64 years were extracted from the STEPS database and enrolled into the study. Four thousand (4466) study participants (62.8%) were females. The mean age of respondents was 35.3 years (SD 12.5), and a third of the respondents were between 25 to 34 years old. Respondent residing in rural area, less educated, self-employed, and married were more represented in the study. The overall prevalence of hypertension in Rwanda was 15.4% (95% CI: 14.6%–16.3%); this was 16.5% (95% CI: 15.1%–18.0%) among males and 14.4% (95% CI: 13.4%–15.5%) among females. The prevalence of hypertension was more than double in those aged 55–64 years as compared to those 44 years and below (38.6%). The Western and Northern Provinces were more affected while prevalence of hypertension was higher in the semi-urban areas (18.6%). Prevalence of hypertension was higher among the separated, divorced and widowed, the less educated, and employed. The lowest rate of hypertension was observed among students (Table 1).

**Table 1:** Distribution of respondents by select socio-demographic characteristics and hypertension rates, 2013

	Men			Women			Both sex			*p*-value
Variable	n	%	95% CI	n	%	95% CI	n		95% CI	
Overall	2650	16.5	15.1-18.0	4466	14.4	13.4-15.5	7116	15.4	14.6-16.3	0.018
Age group										
15–24	560	8.4	6.3–11.0	925	7.4	5.8–9.2	1485	7.9	6.5–9.4	<0.001
25–34	912	18.0	15.6–20.6	1437	8.9	7.5–10.5	2349	13.2	11.9–14.7	
35–44	554	20.0	16.9–23.6	982	18.3	16.0–20.9	1536	19.1	17.2–21.2	
45–54	388	25.0	20.9–29.6	653	30.3	26.9–33.9	1041	27.8	25.2–30.7	
55–64	236	38.1	32.1–44.5	469	41.4	37.0–45.9	705	39.9	36.3–43.7	
Province										
Eastern	636	13.4	10.9–16.4	1028	11.8	10.0–13.9	1664	12.6	8.4–14.3	<0.001
Kigali	282	15.2	11.4–20.0	523	13.5	10.8–16.7	805	14.2	11.9–16.9	
Northern	447	19.1	15.6–23.2	761	16.0	13.6–18.7	1208	17.5	11.4–19.8	
Southern	584	16.5	15.7–19.7	944	13.3	11.4–15.6	1528	14.9	13.2–16.8	
Western	701	18.6	15.8–21.8	1210	17.2	15.1–19.4	1911	17.8	16.1–19.7	
Locality										
Rural	2106	16.0	14.5–17.7	3469	14.2	13.0–15.3	5575	15.0	14.1–16.0	0.082
Semi urban	208	20.8	15.7–27.0	384	16.7	13.2–20.8	592	18.5	15.5–22.0	
Urban	336	17.0	13.3–21.5	613	14.6	12.1–17.6	949	15.7	13.5–18.3	
Level of Education										
No formal school	405	18.4	14.9–22.5	1025	22.2	19.8–24.9	1430	20.8	18.7–23.0	<0.001
Primary school	1813	16.5	14.9–18.3	2849	12.1	10.9–13.3	4662	14.2	13.2–15.3	
Secondary school	371	14.6	11.3–18.6	539	15.3	12.6–18.6	910	15.0	12.7–17.5	
University	61	20.8	12.6–32.4	53	10.5	4.3–23.3	114	16.9	11.1–25.0	
Marrital Status										
Never Married	757	10.3	8.4–12.7	922	8.9	7.2–10.9	1679	9.7	8.3–11.2	<0.001
Married/Cohabitating	1781	21.0	19.1–23.0	2764	15.0	13.7–16.3	4545	17.8	16.7–18.9	
Separated/Divorced/Widowed	110	21.7	14.8–30.5	776	24.8	21.9–28.0	886	24.3	21.5–27.2	
Occupation										
Employed/paid	153	22.8	16.6–30.3	145	12.7	8.0–19.4	298	18.7	14.5–23.8	<0.001
Self-employed	2137	18.3	16.7–20.0	3689	15.6	14.5–16.8	5826	16.9	15.8–17.8	
Student	211	4.6	2.5–8.5	274	5.4	3.3–8.8	485	5.0	3.3–7.4	
Unemployed	146	16.9	11.6–24.0	351	16.1	12.7–20.2	497	16.4	13.4–20.0	
Not mentioned	3	0.0		7	11.5	0.7–70.0	10	6.3	0.5–46.3	
Screening & Awareness										
Previously diagnosed	73	12.8	10.1–15.9	272	33.6	30.2–37.2	345	23.0	20.7–25.4	
Aware	25	34.6	24.1–46.8	80	27.2	22.2–32.9	115	29.3	24.5–34-5	

Among respondents who were hypertensive, 23.0% self-reported that they had been previously diagnosed by a registered physician and 70.7% reported to have never been told by a physician or a health professional that they had high blood pressure. Seventy nine percent of people who had hypertension in the rural areas were undiagnosed as compared to 71.2% in semi-urban/urban areas (Table 1).

### Hypertension and associated risk factors

The prevalence of hypertension was 2 times higher among respondents with BMI more than or equal to 30 kg/m^2^ as compared to those with normal BMI. The descriptive analysis showed that hypertension prevalence was higher among respondents who had a history of smoking, alcohol consumption, and those with low physical activity. The prevalence was also higher among respondents who had high fasting blood glucose or were on medication for diabetes, and those with raised total cholesterol (Table 2).

**Table 2:** Distribution of respondents by hypertension status, common risk factors and sex, Rwanda, 2013

	Men			Women			Both sex			*P*-value
Variables	n	%	95% CI	n	%	95% CI	n		95% CI	
Overall	2650	16.5	15.1-18.0	4466	14.4	13.4-15.5	7116	15.4	14.6-16.3	0.018
Smoking										
Never smoked	1990	15.9	14.4–17.6	3986	13.5	12.5–14.6	5976	14.6	13.7–15.5	<0.001
Past smokers	43	16.4	7.3–32.7	59	29.7	19.2–42.9	102	22.2	14.7–31.9	
Current smokers(non-daily)	156	15.5	10.5–22.3	169	26.2	19.9–33.7	325	19.6	15.4–24.5	
Daily smokers	461	20.4	16.8–24.4	252	22.3	17.5–17.9	713	20.9	17.9–24.1	
Alcohol consumption										
No	841	12.7	10.6–15.1	2257	12.7	11.4–14.1	3098	12.7	11.6–13.9	<0.001
Yes	1809	18.6	16.8–20.5	2209	16.4	14.9–18.0	4018	17.6	16.4–18.9	
Physical activity										
Low	1264	20.1	17.9–22.4	3007	15.0	13.8–16.3	4271	16.9	15.8–18.0	<0.001
High	1386	13.8	12.1–15.8	1459	13.5	11.8–15.3	2845	13.7	12.4–15.0	
Fruit and vegetable consumption										
5 or more fruit/veg/day	23	34.2	16.9–57.0	44	9.9	4.1–21.5	67	19.4	11.6–30.7	0.364
< 5 of fruit/veg on/day	2627	16.4	15.0–17.9	4422	14.5	13.5–15.5	7049	15.4	14.5–16.3	
BMI(kg/m2)										
Underwieght	249	10.2	7.3–14.0	278	11.3	8.2–15.4	527	10.6	8.4–13.3	<0.001
Normal weight	2144	15.9	14.4–17.5	3153	13.1	11.9–14.3	5297	14.5	13.6–15-5	
Overweight	231	27.6	21.9–34.1	815	17.6	15.1–20.3	1046	20.4	18.0–23.1	
Obese	26	46.0	26.2–67.2	220	27.3	21.7–33.6	246	29.9	24.3–36.2	
Blood glucose										
< 5.1 mmol/l	2332	16.6	15–1–18-1	3991	14.3	13.2–15.4	6323	15.4	14.5–16.3	0.0209
5.1–6.1 mmol/l	48	14.7	7.4–27.1	63	15.1	8.2–26.0	111	14.9	9.5–22.6	
> =6.1 or took medication	85	19.9	12.1–30.9	125	25.3	18.4–33.9	210	22.5	17.0–29.2	
Total Cholesterol										
< 5.0 mmol/dl	2544	16.2	14.8–17.7	4228	14.2	13.2–15.3	6672	15.2	14.3–16.0	<0.001
> = 5.0 mml/dl	61	34.2	23.4–49.9	154	21.4	15.7–28.4	215	26.6	20.9–33.4	
HDL Cholesterol										
< 1.03 mmol/dl for men or <1.29 for women	1583	14.0	12.3–15.7	3196	14.0	12.9–15.3	4779	14.0	13.0–15.3	<0.001
> =1.03 mmol/dl for men or > = 1.29 for women	1030	21.2	18.7–23.8	1201	15.9	13.9–18.0	2231	18.9	17.2–20.6	

The multivariate logistic regression showed that increase in age was associated with a linear increase in the risk of having hypertension, being four times more prevalent among respondents aged 55–64 (AOR: 8.02, 95% CI: 5.63–11.42*, p*-value < 0.001) compared to those aged 25–34 years old (AOR: 1.69, 95%CI: 1.25–2.80, *p-*value < 0.001). The risk of having hypertension was respectively 1.30 (95% CI: 1.01–1.67, *p*-value = 0.028,) and 1.24 (95% CI: 1.05–1.44, *p*-value = 0.009) times higher among respondents who reside in semi-urban area and those who consumed alcohol. Females (AOR: 0.75, 95% CI: 0.63–0.88, *p*-value < 0.001,) and students (AOR: 0.45, 95% CI: 0.25–0.80, *p*-value = 0.007,) were less likely to be hypertensive. The risk of having hypertension was 2 times higher among obese respondents (AOR: 3.93, 95% CI: 2.54–6.08, *p*-value < 0.001,) compared to those with normal BMI (AOR: 1.74, 95% CI: 1.30–2.32, *p*-value < 0.001) (Table 3).

**Table 3:** Risk factors associated with hypertension, Rwanda 2013

Variables	Weighted %	UOR	95% CI	*p*-value	AOR	95% CI	*p*-value
Age group							
15–24	7.9	1			1		
25–34	13.2	1.79	1.42–2.25	0.000	1.69	1.25–2.80	0.001
35–44	19.1	2.77	2.19–3.50	0.000	2.53	1.84–3.48	<0.001
45–54	27.8	4.52	3.56–5.74	0.000	4.37	3.14–6.10	<0.001
55–64	39.9	7.80	6.08–10.00	0.000	8.02	5.63–11.42	<0.001
Gender							
Male	16.5	1			1		
Female	14.4	0.85	0.74–0.97	0.018	0.75	0.63–0.88	<0.001
Locality							
Rural	15.0	1			1.0		
Semi urban	18.5	1.29	1.02–1.61	0.031	1.30	1.01–1.67	0.040
Urban	15.7	1.05	0.86–1.28	0.614	1.01	0.79–1.28	0.940
Level of Education							
No formal schoool	20.8	1			1.0		
Primary school	14.2	0.63	0.54–0.74	0.000	0.92	0.77–1.10	0.354
Secondary school	15.0	0.67	0.53–0.84	0.001	1.22	0.93–1.60	0.151
University and High	16.9	0.77	0.47–1.28	0.322	1.01	0.58–1.78	0.962
Marital Status							
Never Married	9.7	1			1		
Married/Cohabitating	17.8	2.02	1.68–2.42	0.000	0.87	0.67–1.13	0.298
Separated/Divorced/Widowed	24.3	2.99	2.40–3.75	0.000	0.98	0.72–1.34	0.905
Occupation							
Employed/paid	18.7	1			1		
Self-employed	16.9	0.88	0.64–1.20	0.433	0.80	0.56–1.15	0.223
Student	5.0	0.23	0.14–0.38	0.000	0.45	0.25–0.80	0.007
Unemployed	16.4	0.85	0.58–1.26	0.430	0.84	0.55–1.30	0.441
Not mentioned	6.3	0.29	0.03–2.44	0.257	-		-
Smoking							
Never smoked	14.6	1			1		
Past smokers	22.2	1.67	1.01–2.74	0.044	1.05	0.60–1.86	0.853
Current smokers(non-daily)	19.6	1.42	1.06–1.91	0.018	0.84	0.60–1.17	0.301
Daily smokers	20.9	1.54	1.26–1.90	0.000	0.83	0.65–1.06	0.145
Alcohol consumption							
No alcohol consuption	12.7	1			1		
Alcohol consumption	17.6	1.47	1.28–1.68	0.000	1.24	1.05–1.44	0.009
Physical activity							
Low	16.9	1			1		
High	13.7	0.78	0.68–0.90	0.000	1.01	0.86–1.18	0.892
Fruit and vegetable consumption							
5 or more fruit/veg/day	19.4	1			-		-
< 5 servings fruit/veg /day	15.4	0.75	0.41–1.37	0.358	-		-
BMI (kg/m2)							
< 18.5	10.6	1			1		
18.5–24.9	14.5	1.43	1.09–1.87	0.009	1.74	1.30–2.32	0.000
25–29.9	20.4	2.16	1.60–2.92	0.000	2.60	1.87–3.63	0.000
≥ 30	29.9	3.59	2.45–5.26	0.000	3.93	2.54–6.08	0.000
Blood Glucose							
< 5.1 mmol/l	15.4	1		1	1		
5.1–6.1 mmol/l	14.9	0.96	0.58–1.60	0.885	0.76	0.46–1.28	0.31
> =6.1 or took medication	22.5	1.60	1.12–2.28	0.009	1.26	0.85–1-87	0.242
Total Cholesterol							
< 5.0 mmol/dl	15.2	1		1	1		
> = 5.0 mml/dl	26.6	2.03	1.46–2.82	0.000	1.27	0.88–1.84	0.193
HDL Cholesterol							
Low	14.0	1		1	1		
Normal HDL Cholesterol	18.9	1.43	1.24–1.64	0.000	1.16	1.00–1.36	0.050

## Discussion

This study provided national population-based and age-adjusted estimates of hypertension in Rwanda, which showed an overall prevalence of 15.3%, among the population aged 15 to 64 years with males being more affected than females. This prevalence was about 6 times higher than what was reported through hospital-based data [14], but similar to the findings of a previous study in Bugesera district [16]. High prevalence was observed among the less educated and respondents who were not in union with their spouses (separated, divorced and widowed). More than three quarter of the hypertensive persons had never been informed about their high blood pressure, hence a lower level of awareness. Increasing age, living in semi-urban area, alcohol consumption and raised BMI were significantly associated with hypertension which compared favorably to the findings of other studies in and outside the region [32–41].

The prevalence of hypertension observed in this study is lower than what has been documented by similar studies conducted in Sub-Saharan African countries nevertheless we consider this prevalence as high. The low level of awareness of study participants observed in these findings might perhaps be due to inadequate public health interventions for prevention and control of hypertension. Health programs have for long time focused on communicable diseases rather than non-communicable conditions. Moreover, the enhanced surveillance of NCDs including hypertension was not yet fully integrated in the existing health system in Rwanda. Similar findings have also been reported across and outside the continent [10, 42–45]. However, contrasting findings of high level of awareness and control of hypertension was also observed in developed countries [46, 47].

The high prevalence of hypertension among less educated was documented by other studies [48–51] and could be attributed to the lack of appropriate health information and poor health education programs which normally improve the knowledge of the population and enhance the level of awareness of chronic conditions [52, 53]. Furthermore, uneducated people are less likely to understand risk factors related to diseases induced by lifestyle and thus unable to change their behavior and adopt positive practices [54, 55]. Although the high prevalence of hypertension was evident among less educated in our study, contrasting findings have been reported [56–58].

The increased prevalence of hypertension among separated, divorced and widowed could be due to stress induced by the absence of psychosocial and economic support from a spouse. On the other hand, separated, divorced and widowed are more likely to lose interest in life, therefore engage more in high-risk health behaviors such as smoking, alcohol consumption, lack of exercises and change in diet [59–63].

The socio-economic transition and urbanization observed in Rwanda which results in lifestyle, dietary changes and unhealthy behaviors may explain the high risk of hypertension in semi-urban area observed in this study. The impact of urbanization on lifestyle and health has been reported by other authors [64–68]; however our findings on association between hypertension and semi-urban residence were inconsistent with what was reported in a few low and middle income countries [69–72].

### Study limitations

This study has three main limitations. First, the original study was a cross-sectional survey, thus the simultaneous collection of information on high blood pressure and its risk factors could have clouded the association between a risk factor and an outcome. Therefore, only an association and not causation can be inferred.

Second, the prevalence of hypertension may have been over or under-estimated because the three blood pressure measurements were performed on one occasion only. However, the large sample size, the use of standardized and tested methodologies, tools and high response rate observed in the study increased its representativeness and minimized this limitation.

Third, we were unable to explore the contribution of other risk factors such as unhealthy diet, salt intake, and psychosocial stress on the prevalence of hypertension because the related information were not collected during primary data collection.

## Conclusions

The findings of this study show that the prevalence of hypertension is high in Rwanda. However, the level of awareness about the disease is low. The study identified age, overweight and obesity, alcohol consumption, residence in semi-urban area as the risk factors associated with hypertension. These findings and those of other similar studies should be used to synthesize appropriate evidence-based policies for the prevention and control of hypertension in the country. Prevention strategies including the integration of hypertension surveillance system into national disease surveillance system, conducting targeted screening of high-risk groups, and treatment (where indicated) of all persons attending health facilities are recommended. Measures aimed at reducing harmful use of alcohol especially among the high-risk group are also proposed. Furthermore, stakeholders should put in place strong health promotion and awareness creation aimed at addressing identified risk factors. Further studies on the association of hypertension and other factors such as psychosocial stress, salt and oil intake which were not examined in this study are essential to inform NCD program managers on the exhaustive risk factors of hypertension in the country and inform development of prevention and control strategies.

## Additional files

Additional file 1: STEPS Survey Sampling Flowchart. (DOCX 172 kb)

Additional file 2: Variables Re-categorized from the Original Dataset. (DOCX 13 kb)

## Abbreviations

AOR: Adjusted Odds Ratio
BMI: Body Mass Index
CDC: Center for Disease Control
CHWs: Community Health Workers
CI: Confidence Interval
HDL: High Density Lipoproteins
HIV/AIDS: Human Immunodeficiency Virus/Acquired Immunodeficiency Syndrome
HTN: Hypertension
LMIC: Low Middle Income Countries
MOH: Ministry of Health
NCDs: Non Communicable Diseases
NISR: National Institute of Statistics of Rwanda
PDA: Personal Digital Assistant
PPS: Probability Proportional to Size
RBC: Rwanda Biomedical Center
SD: Standard Deviation
WHO: World Health Organization

## Footnotes

1 The Kish sampling method was developed by Leslie Kish (1910–2000); itis built into the PDAs which were used to select eligible respondents in the households when more than one household members are eligible for the interview. Once a household is selected, the interviewer creates a list (sampling frame) of all the persons in the household that are eligible for the interview process. This list includes the name of the person, their gender, their relationship to the head of the household and their age. Once the listing is done, each eligible member is assigned a unique number. Then using a randomized response table a particular member is chosen for the interview.
