# Supplementary material for: A population-based national estimate of the prevalence and risk factors associated with hypertension in Rwanda: implications for prevention and control
Source: BMC Public Health. 2017 Jul 10;18:2. doi: 10.1186/s12889-017-4536-9 (PMC5504833; doi:10.1186/s12889-017-4536-9)
Supplement: Supplementary file 1 — STEPS Survey Sampling Flowchart. (DOCX 172 kb) [file 12889_2017_4536_MOESM1_ESM.docx]

Additional file 1: Sampling flow chart
