# Supplementary material for: A population-based national estimate of the prevalence and risk factors associated with hypertension in Rwanda: implications for prevention and control
Source: BMC Public Health. 2017 Jul 10;18:2. doi: 10.1186/s12889-017-4536-9 (PMC5504833; doi:10.1186/s12889-017-4536-9)
Supplement: Supplementary file 2 — Variables Re-categorized from the Original Dataset. (DOCX 13 kb) [file 12889_2017_4536_MOESM2_ESM.docx]

**Additional file 2: Variables Re-categorized from the Original Dataset**

| **Education** | **Marital Status** | **Occupation** | **Alcohol consumption** | **Physical activity** | **BMI (kg/m^2^)** |
| --- | --- | --- | --- | --- | --- |
| No formal school | Single | Employed/paid | No alcohol consumption | High | <18.5 |
| Primary school | Married/Cohabitating | Self-employed | Alcohol consumption | Low | 18.5-24.9 |
| Secondary school | Separated/Divorced/Widowed | Student |  |  | 25-29.9 |
| University |  | Unemployed |  |  | ≥ 30 |
